# The role of the medial plate for Pauwels type III femoral neck fracture: a comparative mechanical study using two fixations with cannulated screws

**DOI:** 10.1186/s40634-019-0187-3

**Published:** 2019-05-02

**Authors:** Vincenzo Giordano, Danilo Diniz Alves, Roger Pletsch Paes, Arthur Bonfim Amaral, Marcos Giordano, William Belangero, Anderson Freitas, Hilton A. Koch, Ney Pecegueiro do Amaral

**Affiliations:** 1 Serviço de Ortopedia e Traumatologia Prof. Nova Monteiro, Hospital Municipal Miguel Couto, Rua Mário Ribeiro 117, Leblon, Rio de Janeiro, RJ 22430-160 Brazil; 2 Núcleo Especializado de Ortopedia e Traumatologia, Clínica São Vicente, Rio de Janeiro, Brazil; 3 Serviço de Traumato-Ortopedia, Hospital de Força Aérea do Galeão, Rio de Janeiro, Brazil; 4 Departamento de Ortopedia e Traumatologia, Universidade Estadual de Campinas, Campinas, Brazil; 5 Serviço de Quadril, Hospital Ortopédico e Medicina Especializada – HOME, Brasília, Brazil; 6 Departamento de Radiologia, Universidade Federal do Rio de Janeiro, Rio de Janeiro, Brazil

**Keywords:** Femoral neck fracture, Pauwels classification, Biomechanical testing, Medial plate, Multiple cannulates screws

## Abstract

**Background:**

The biomechanical behavior of Pauwels type III fractures should be taken into consideration when performing internal fixation, since this repair should resist the shear force inherent in the vertical fracture line to the greatest extent possible. Recently, the use of a small fragment plate on the medial face of the femoral neck has been proposed by some authors, with satisfactory initial results. In the current study we analyze the mechanical role a medial plate used as a buttress plate for Pauwels type III femoral neck fractures, comparing the resistance of two fixation configurations using three cannulated screws.

**Methods:**

Pauwels type III fractures were simulated in synthetic bones models and two groups were created, one of those using two parallel screws at the bottom of the femoral neck and the third screw crossing the fracture horizontally (G1), and the other fixed in the same arrangement as G1, but with the addition of a medial side plate at the apex of the fracture (G2).

The constructs were subjected to axial loading until catastrophic failure.

**Results:**

The addition of a medial plate buttressing the femoral neck increased significantly the resistance to maximum loading (*p* = 0.003).

**Conclusion:**

Use of a medial buttress plate results in a mechanically superior construction for Pauwels type III fractures fixed with multiple cannulated screws.

**Lebel of evidence:**

Level IV. Biomechanical comparative study.

## Background

Fixation of femoral neck fractures is associated with a higher incidence of complications than any other fracture (Estrada et al., 2002). Initial deviation of the fracture occurs in up to two thirds of cases, leading to complications such as non-union, osteonecrosis (ON), and collapse of the femoral head, mainly through interruption of vascular supply. This is more critical in the young adult population, where preservation of the femoral head is the rule (Estrada et al., 2002; Panteli et al., 2015; Damany et al., 2005). Beyond initial deviation, other characteristics such as posterior fragmentation of the femoral neck and Pauwels type III fracture increase the risk that complications will occur and that additional procedures will be necessary (Panteli et al., 2015). Careful evaluation and preoperative planning are important, because fixation failure in young adults is associated with high morbidity and is difficult to solve (Estrada et al., 2002; Panteli et al., 2015; Damany et al., 2005; Shen et al., 2016).

The occurrence of more vertical fractures in the femoral neck, classified as Pauwels type III fracture, is very frequent in young adults, usually after high-energy trauma (Shen et al., 2016). The geometry of the fracture line has been shown to depend on the energy of the injury and patient age (Panteli et al., 2015; Basso et al., 2012). Shear forces are dominant in this type of fracture, resulting in the deviation and collapse of the proximal end of the femur in varus (Shen et al., 2016). The biomechanical behavior of Pauwels type III fractures should be taken into consideration when performing internal fixation, since this repair should resist the shear force inherent in the vertical fracture line to the greatest extent possible (Panteli et al., 2015; Shen et al., 2016; Gümüstas et al., 2014). Several studies have suggested that the ideal osteosynthesis for Pauwels type III femoral neck fractures should consider this characteristic, although no consensus has been reached on the best type of fixation.

Today, fixation using three cannulated screws with diameters of larger than 6.0-mm is most commonly recommended to treat femoral neck fractures, since it provides the best axial and torsional stiffness, resulting in improved failure strength (Asnis, 1985; Augat et al., 2019; Yang et al., 2013; Zdero et al., 2010). However, in Pauwels type III fractures this type of construction generates not only compressive forces but also shear forces, which increases the risk of deviation between the fragments and collapse in varus (Shen et al., 2016). Adverse outcomes have been observed in 20% to 48% of patients who undergo this type of fixation (Filipov, 2011). New arrangements employing multiple cannulated screws or a sliding hip screw system have been studied in the literature to manage this fracture pattern, although no consensus has been reached (Panteli et al., 2015; Augat et al., 2019; Filipov, 2011; Luttrell et al., 2014; Nowotarski et al., 2012). More recently, the use of a small fragment plate on the medial face of the femoral neck has been proposed by some authors, with satisfactory initial results (Mir & Collinge, 2015; Ye et al., 2017). In theory, by adding a medial plate to buttress a Pauwels type III would transform shearing forces in compressive forces.

In this present study, the authors compared two forms of fixation for Pauwels type III fractures using cannulated screws: two parallel screws at the bottom of the femoral neck with a third screw horizontally crossing the fracture (widely known as the Pauwels screw), and the use of a small fragment plate in the medial portion of the femoral neck combined with cannulated screws in the same arrangement as described above. The objective of this study was to use an experimental model with synthetic bones to determine the biomechanical stability of these two arrangements in fixation for Pauwels type III femoral neck fractures. Hypothetically we assume that the addition of a medial plate displays higher stability than a construction with multiple cannulated screws.

## Methods

A total of 10 synthetic bone models of the right femur (model 2240, Synbone, Switzerland) were used; these were 465.0 mm long, with a condylar width of 86.0 mm, femoral neck angle of 135° and 15° of anteversion, femoral head diameter of 48.0 mm, and medullary canal diameter of 9.5 mm. The bones were divided into two equal groups: group 1 (G1), which had Pauwels type III fractures fixated using two parallel screws at the bottom of the femoral neck and the third screw crossing the fracture horizontally (the Pauwels screw), and group 2 (G2), which had Pauwels type III fractures fixated in the same arrangement as G1, but with the addition of a medial side plate at the apex of the fracture.

## Preparing the testing specimens

A goniometer was used to create osteotomy lines at a 70° angle on the testing specimens in the middle third of the femoral neck, reproducing a Pauwels type III fracture (Shen et al., 2016). Before the osteotomy was created, the guide wires for the cannulated screws were introduced using fluoroscopy according to the fixation assembly used in the two experimental groups in order to facilitate reduction and fixation after the bone was cut. The wires were removed and an oscillating saw was used to cut the femoral necks according to the osteotomy lines which had been drawn, working from back to front until just before the anterior cortex was reached. The osteotomy was completed manually so that after reduction there would be anatomical contact with the anterior cortex of the synthetic bone.

Next, the osteotomy was anatomically reduced and the wires were repositioned in the holes which were made initially. A cannulated drill was used only in the lateral cortex of the femur and 7.0 mm self-tapping cannulated screws were introduced over the guide wires until they reached 5.0 mm from the articular surface of the femoral head. Washers were not used. Interfragmentary compression of the osteotomy was created using the two inferior screws, and subsequently the Pauwels screw was introduced. This latter cannulated screw was positioned centrally in the plane of the femoral neck, located in the posterior and anterior neck of the femur (Gurusamy et al., 2005).

**Fig. 1:**
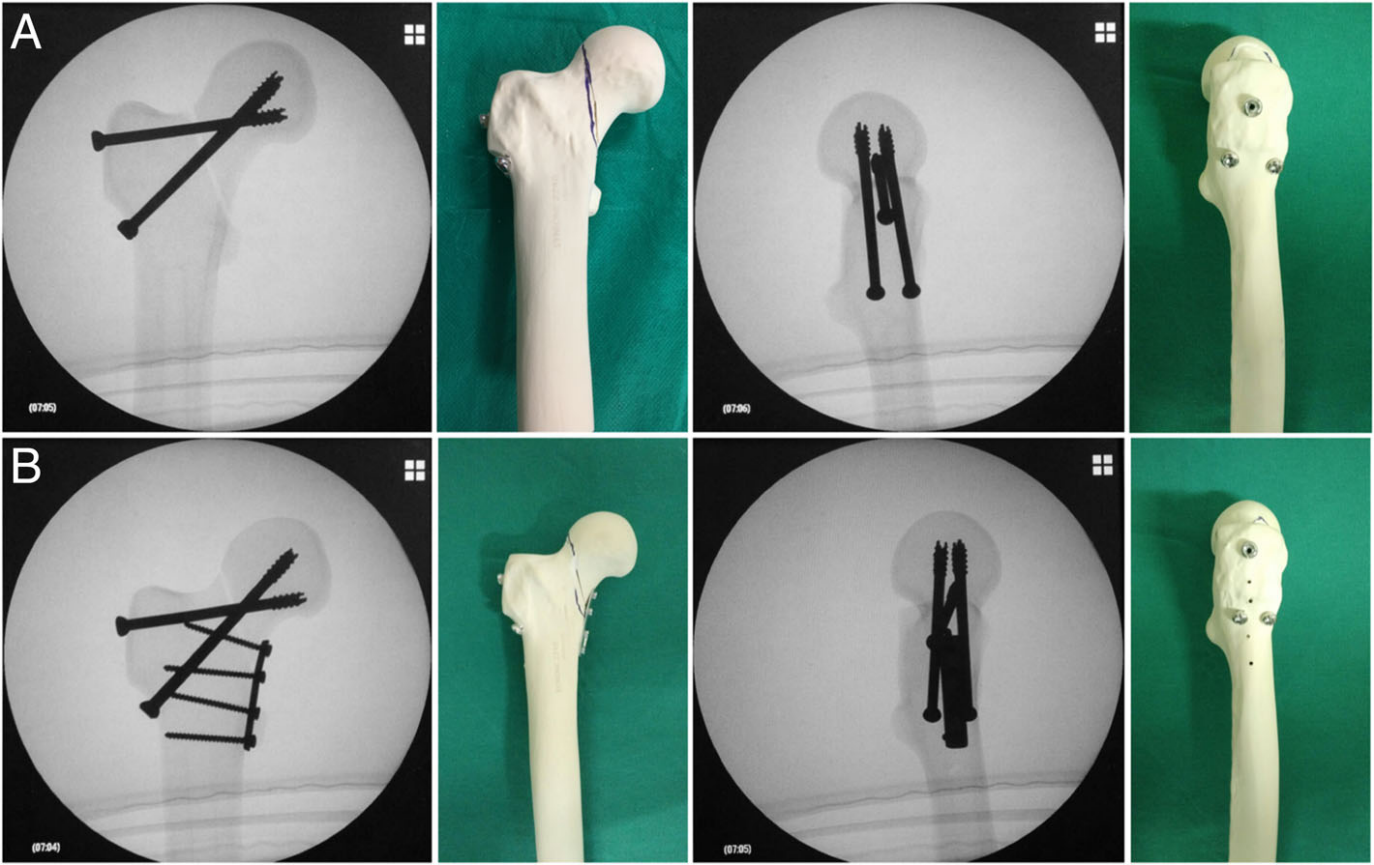
**a**. Group 1 - Assembly with two parallel cannulated screws in the inferior portion of the femoral neck + the Pauwels screw: fluoroscopy in the coronal and sagittal planes, and photographs of one of the specimens prior to testing, anterior and lateral views. Note the parallel arrangement of the two inferior screws in the coronal and sagittal planes. Also note how the Pauwels screw occupies the central space of the femoral neck in the sagittal plane; **b**. Group 2 - Assembly with two parallel cannulated screws in the inferior portion of the femoral neck + the Pauwels screw + medial plate: fluoroscopy in the coronal and sagittal planes, and photographs of one of the specimens prior to testing, anterior and lateral views. Note the parallel arrangement of the two inferior screws in the coronal and sagittal planes. Also note how the Pauwels screw occupies the central space of the femoral neck in the sagittal plane. Observe that only 1 of the 4 screws of the plate was placed proximally to the osteotomy site – this was done to reproduce what normally occurs in the clinical situation, where it is almost impossible in the majority of patients to position the plate in a complete medial position – it is more anteromedial to the lesser trochanter – and also to put it more proximally, due to the inferior capsule

In the G2 group, after the three cannulated screws were placed, a four-hole side plate was placed on the medial face of the femoral neck with 3.5mm cortical screws. Next, anatomic reduction was confirmed visually and using radioscopy (Fig. 1).

## Mechanical testing

For the mechanical test, the specimens were cut in the shaft region, resulting in a final size of 200.0 mm, according to the protocol described previously by the authors (Giordano et al., 2018).

### Testing equipment

We used a Flextest 40 MTS model 810 device (*Materials Testing System*, Eden Prairie, MN, USA) with a 100.0 kN capacity, using a 10.0 kN capacity load cell calibrated and measured by the Mechanical Testing Laboratory in the Department of Manufacturing and Materials Engineering at the School of Mechanical Engineering.

### Load application system

**Fig. 2:**
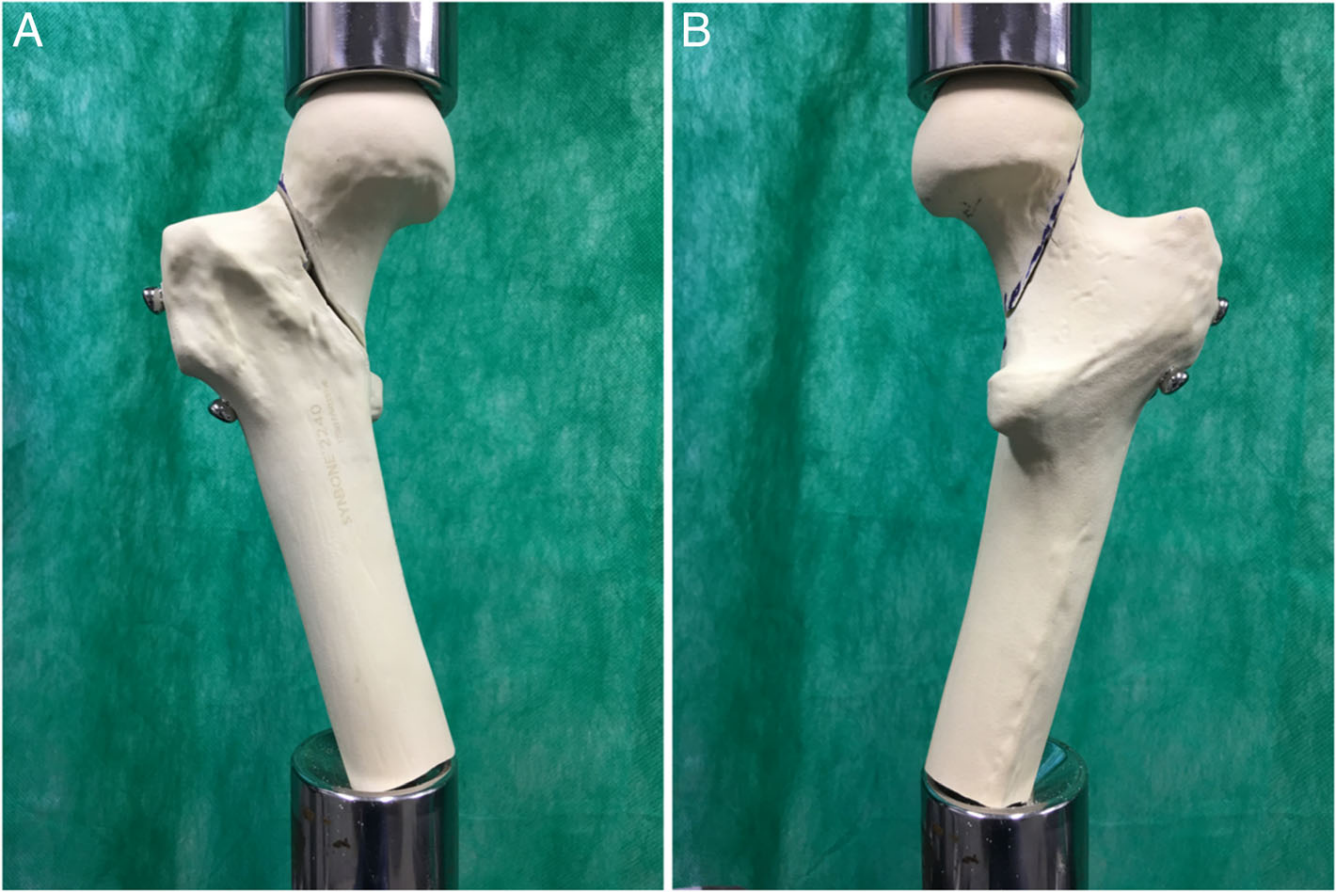
Group 1 - Assembly with two parallel cannulated screws in the lower portion of the femoral neck + the Pauwels screw in one of the specimens prior to mechanical testing, anterior (**a**) and posterior (**b**) views

The specimens were tested longitudinally in an upright position, with a 15° inclination (Fig. 2). Testing for the two experimental fixation groups was conducted according to the following steps:

Step 1: until displacement of 5.0 mm was achieved, to measure the load and the angle of rotation for the femoral head at this point. After measuring, the test was resumed.

Step 2: Until the failure of fixation, when the load was measured.

## Statistical analysis

Descriptive analysis of the data observed (expressed as measures of central tendency and dispersion by fixation group) was presented in the form of tables to verify whether the existence of significant difference in maximum load between the groups.

The inferential analysis consisted of the Kruskal-Wallis ANOVA and Dunn’s multiple comparisons test (non-parametric tests) to verify the existence of significant difference in the maximum load between the groups (Hollander & Wolfe, 1999; Dunn, 1964).

Statistical analysis was performed using SPSS software version 20.0, and a 5% significance level was adopted.

## Results

The biomechanical testing found different applied load measurements for the two groups.

Tables 1 and 2 present the results obtained in step 1, when load was applied until a 5.0 mm displacement was achieved; at this point load was measured (Table 1) along with the rotation of the femur head at this point (Table 2). The tables show that G1 (two parallel cannulated screws at the bottom of the femoral neck + Pauwels screw) resisted a smaller load before 5.0 mm displacement was achieved in comparison with G2 (two parallel cannulated screws at the bottom of the femoral neck + Pauwels screw + medial plate). Rotation of the femoral head was greater in G2 than in G1.

**Table 1:** Load values in newtons (N) for 5.0 mm of displacement

Specimen	G1 (2 parallel screws + Pauwels screw)	G2 (2 parallel screws + Pauwels screw + medial plate)
1	703	973
2	739	1204
3	960	1178
4	373	1393
5	527	1235
***Mean***	***660***	***1197***
***SD***	***223***	***150***

Source: Serviço de Ortopedia e Traumatologia Prof. Nova Monteiro, 2017

**Table 2:** Values for rotational deviation of the femoral head with 5.0 mm of displacement

Specimen	G1 (2 parallel screws + Pauwels screw)		G2 (2 parallel screws + Pauwels screw + medial plate)	
	Rotation (mm)	Rotation (degrees)	Rotation (mm)	Rotation (degrees)
1	0.00	0.00	0.00	0.00
2	0.00	0.00	0.10	0.38
3	0.30	1.14	0.30	1.14
4	0.00	0.00	1.50	5.68
5	0.00	0.00	1.40	5.31
***Mean***	***0.06***	***0.23***	***0.66***	***2.50***
***SD***	***0.13***	***0.51***	***0.73***	***2.77***

Source: Serviço de Ortopedia e Traumatologia Prof. Nova Monteiro, 2017

**Fig. 3:**
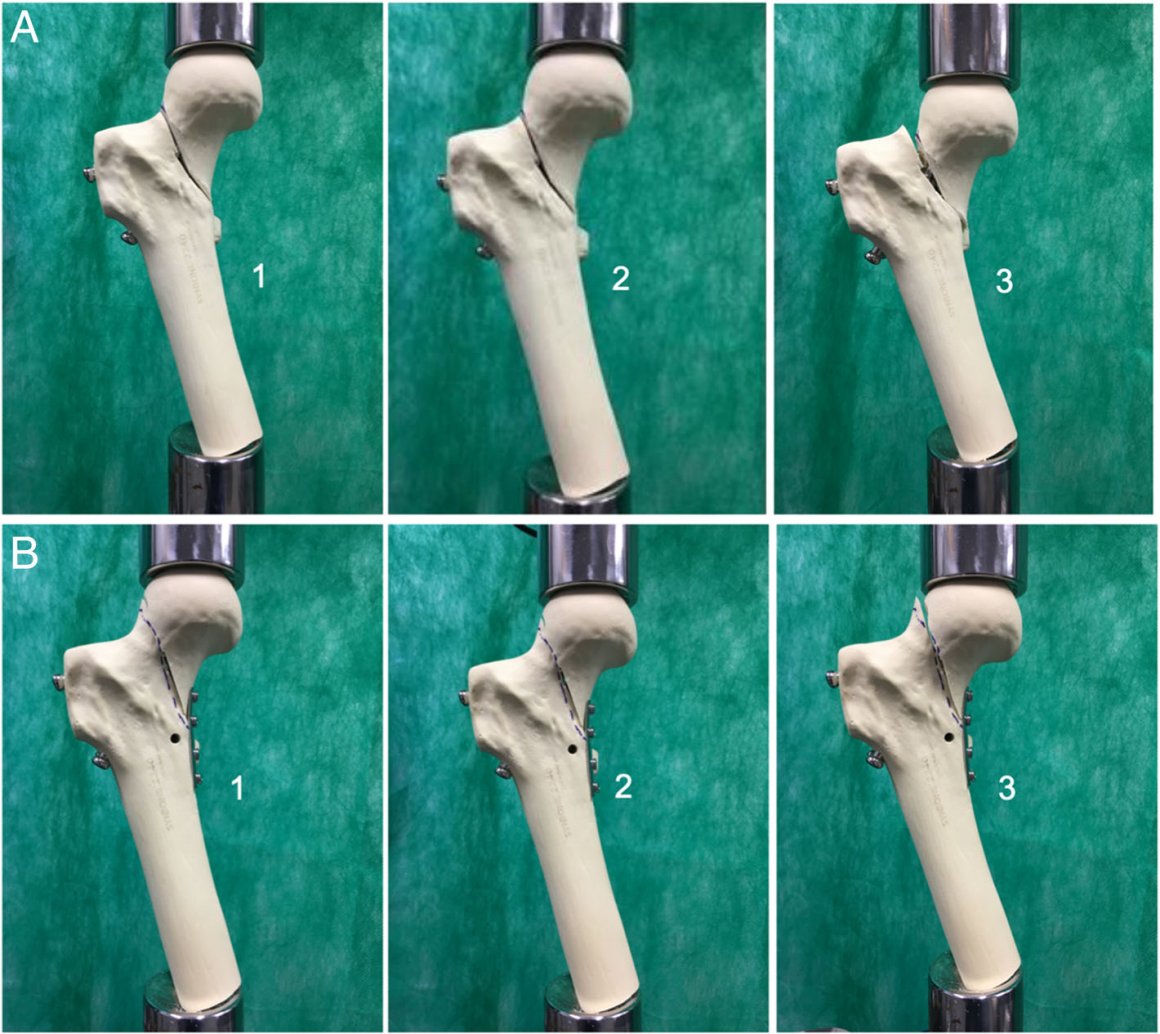
**a**. Group 1 specimen with two parallel cannulated screws in the lower portion of the femoral neck + Pauwels screw, anterior view: (1) prior to testing, (2), after step 1, and (3) after failure in step 2. Note that there was virtually no change after step 1, but step 2 led to varus deviation and shearing of the femoral neck. Note the protrusion of the fixation screws, particularly the inferior screws; **b**. Group 2 specimen with two parallel cannulated screws in the lower portion of the femoral neck + Pauwels screw + medial plate, anterior view: (1) prior to testing, (2) after step 1, and (3) after failure in step 2. Mild rotational deviation occurred in four of the five specimens after step 1. In step 2, a slight diastasis was observed in the superior region of the femoral neck, but no varus deviation or shearing occurred. Note that the inferior fixation screws protruded less after testing than those in the G1 specimens

Tables 3 and 4 present the results obtained in step 2, when load was applied until failure was reached and the load measurement was obtained. In terms of rigidity and resistance to support maximum load, we found that G1 (two parallel cannulated screws at the bottom of the femoral neck + Pauwels screw) was less rigid and resistant than G2 (two parallel cannulated screws at the bottom of the femoral neck + Pauwels screw + medial plate). Figure 3 shows examples of specimens from each test group.

**Table 3:** Rigidity values, in N/mm

Specimen	G1 (2 parallel screws + Pauwels screw)	G2 (2 parallel screws + Pauwels screw + medial plate)
1	140.6	194.6
2	147.8	240.8
3	192.0	235.6
4	74.6	278.6
5	105.4	247.0
***Mean***	***132.0***	***239.0***
***SD***	***45.0***	***30.0***

Source: Serviço de Ortopedia e Traumatologia Prof. Nova Monteiro, 2017

**Table 4:** Maximum load, in Newtons (N)

Specimen	G1 (2 parallel screws + Pauwels screw)	G2 (2 parallel screws + Pauwels screw + medial plate)
1	1286	1238
2	1146	1747
3	1437	1689
4	922	1878
5	784	1650
***Mean***	***1115***	***1640***
***SD***	***265***	***241***

Source: Serviço de Ortopedia e Traumatologia Prof. Nova Monteiro, 2017

**Fig. 4:**
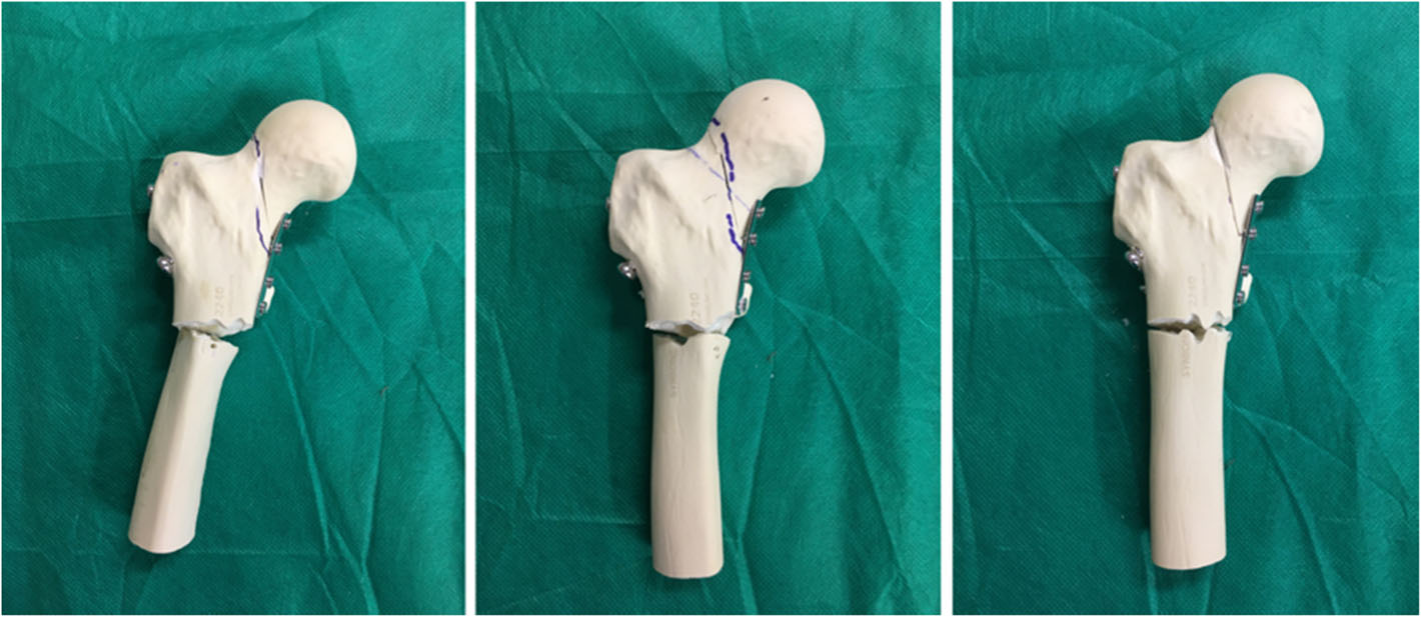
Group 2, anterior view of three of the five test specimens used, with two parallel cannulated screws in the lower portion of the femoral neck + Pauwels screw + medial plate, showing the means of failure in this fixation method

In G2, the experiment was continued in order to observe the means of failure of the medial plate, but this did not occur. Instead, in 3 of 5 test specimens a transverse fracture occurred in the subtrochanteric region just below the medial plate (Fig. 4).

**Fig. 5:**
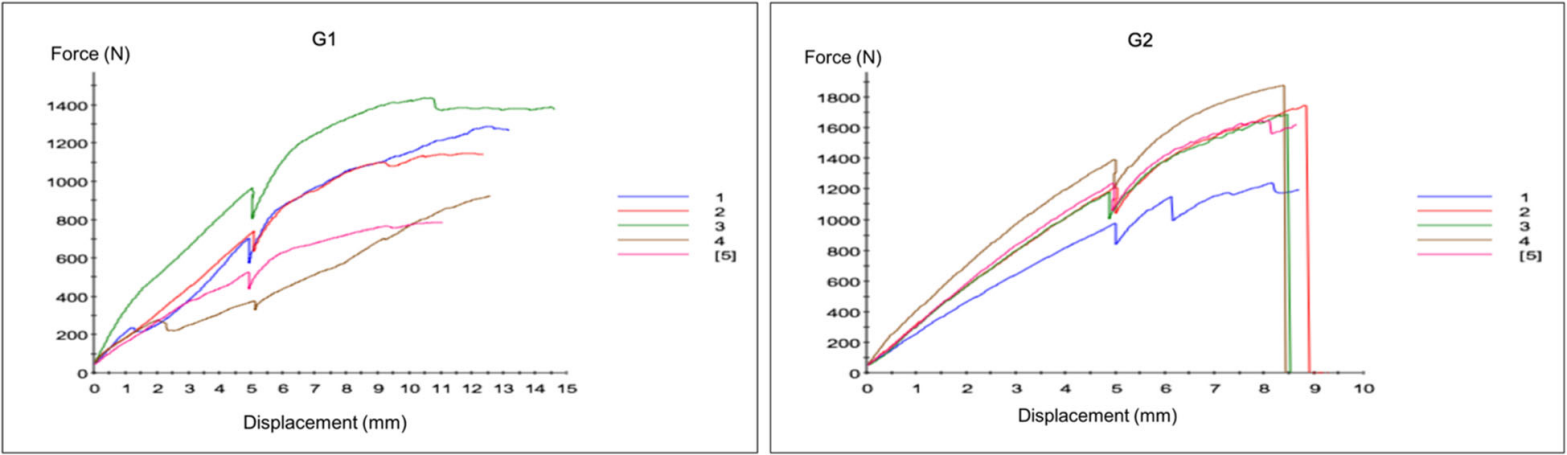
**a**. Group 1 (assembly with two parallel cannulated screws at the bottom of the femoral neck + Pauwels screw): force versus displacement curves for the five specimens analyzed; **b**. Group 2 (assembly with two parallel cannulated screws at the bottom of the femoral neck + Pauwels screw + medial plate): force versus displacement curves for the five specimens analyzed

**Fig. 6:**
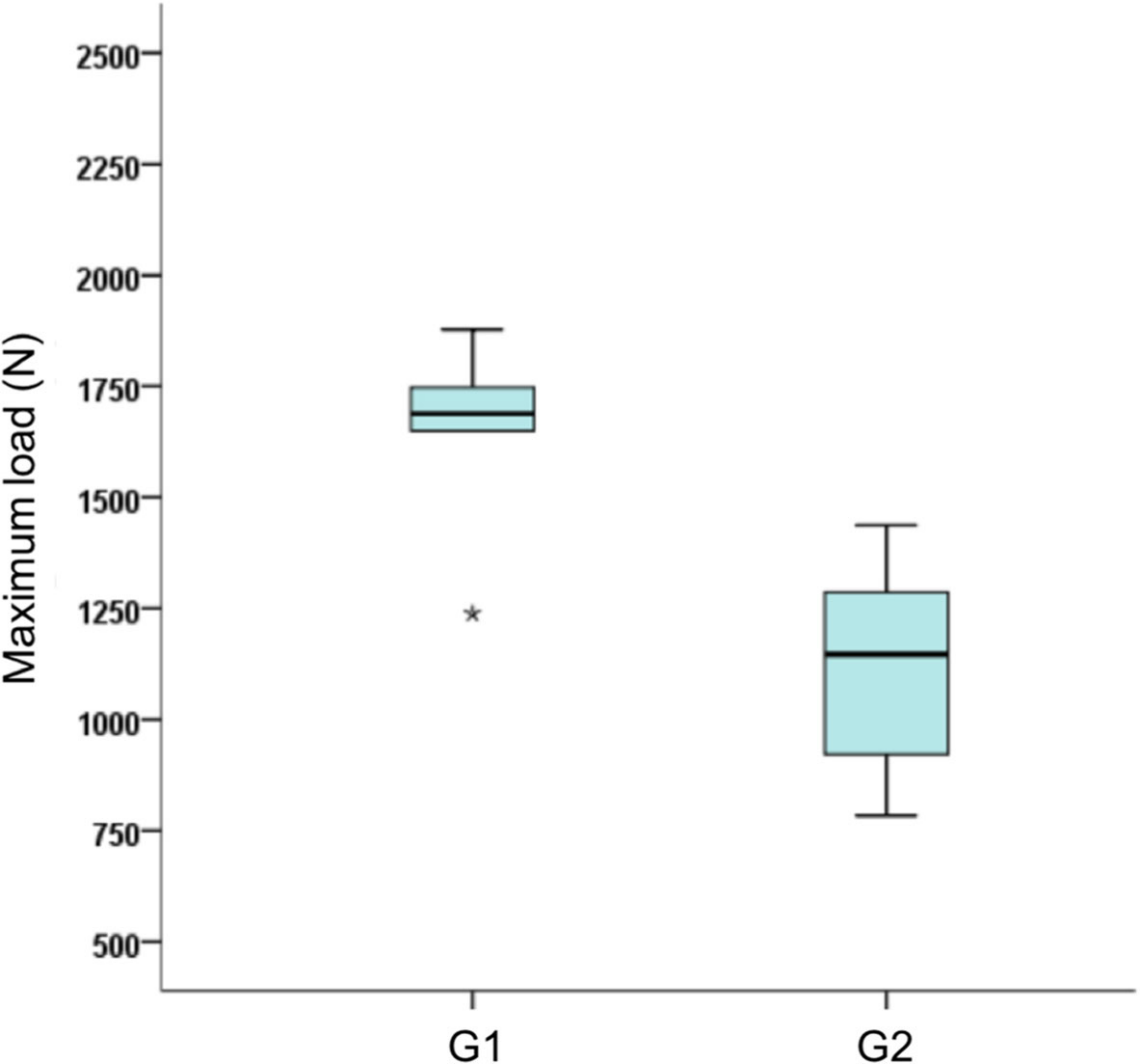
Maximum load (in newtons) by group. The central bar corresponds to the median (2nd quartile) and the bottom and top bars in the box correspond to the 1st (Q1) and 3rd quartiles (Q3), respectively. The top and bottom vertical bars express dispersion of the distribution and the asterisk (*) corresponds to outliers beyond the expected limits

The images in Figs. 5 and 6 represent the force versus displacement curves for groups 1 and 2, respectively in the five specimens which comprised each group.

The data obtained from the biomechanical testing showed statistically significant superiority in terms of strength and stability for the group which combined fixation with the medial plate (G2) when subjected to an axial load. Table 5 provides a complete descriptive of the maximum load (in newtons) according to the fixation groups and the corresponding descriptive level (*p* value) for the Kruskal-Wallis ANOVA.

**Table 5:** Descriptive analysis of maximum load (in newtons) by group

Group	n	Mean	SD	Median	IQR	Min	Max	*p value* ^*a*^	≠ significant ^b^
G1	5	1115	265	1146	853–1362	784	1437	0.003	
G2	5	1640	241	1689	1444-1813	1238	1878		G2 ≠ G1

Source: Serviço de Ortopedia e Traumatologia Prof. Nova Monteiro, 2017

*SD* standard deviation, *IQR* interquartile range (Q1–Q3)

^a^Kruskal-Wallis ANOVA

^b^Dunn’s multiple comparisons text, at 5% significance

Although the mean and standard deviation values are presented in Table 1, the median and interquartile range (Q1–Q3) are more suitable for expressing the maximum load of the groups because they are appropriate measures for data that do not have normal distribution (like the non-parametric tests and box plot), as shown in Fig. 9. A significant difference was found between the fixation groups for the maximum load using the Kruskal-Wallis ANOVA (*p* = 0.003).

## Discussion

Combining a buttress plate in the medial region of the femur neck with cannulated screws improves the mechanical resistance of fixation in Pauwels type III femoral neck fractures. In mechanical tests replicating shear force (a component of the vector of body weight), we found that the assembly using two parallel cannulated screws at the bottom of the femoral neck and the Pauwels screw associated with a medial plate at the fracture apex did not fail in varus or shear, which are typical deviations in this fracture pattern. After an average maximum load of 1640 N, the mode of failure was observed, namely the opening of the upper part of the femoral neck; in the following step, continued deforming force led to subtrochanteric fracture in the peri-implant area in all 3 of 5 specimens.

Mir and Collinge hypothesized that the plate positioned on the medial vertex which characterizes Pauwels type III femoral neck fractures could act as a buttress, resisting shear forces and transforming them into compression forces (Mir & Collinge, 2015). These authors believe that this behavior would theoretically reduce the rate of complications related to the secondary deviations classically found in this fracture pattern. Ye et al. observed 89% consolidation using this assembly in treating 28 patients with Pauwels type III femoral neck fractures, with no cases of avascular necrosis, and two cases of consolidation with shortening of the femoral neck (Ye et al., 2017). Although they call attention to the short follow-up period (average of 13.6 months), the preliminary results were very favorable for using this arrangement in more unstable femoral neck fracture patterns in young adult patients.

The use of cannulated screws only (in the assembly tested in G1 of this experiment) appears to be insufficient for Pauwels type III fractures, although it has been demonstrated superior to other constructions which only use parallel screws on the femoral neck (Panteli et al., 2015; Shen et al., 2016; Gümüstas et al., 2014; Nowotarski et al., 2012; Noda et al., 2015). Luttrel et al. conducted a horizontal study with 247 orthopedists present at the annual meeting of the Orthopaedic Trauma Association (OTA) and found that 28% of these professionals preferred to use an assembly with two cannulated screws parallel to the axis of the femoral neck and another screw outside this axis (the Pauwels screw) to treat Pauwels type III fractures (Luttrell et al., 2014). Fifty-eight per cent of these physicians based their decisions on the fact that this assembly is “more biomechanically stable”, 9.5% stated it presents “fewer complications”, and 8% stated this arrangement was “technically easier”. Only 48% of these surgeons agreed that this assembly is clearly supported by the literature (Luttrell et al., 2014). Indeed, even though this technique shows good results, few studies to date have corroborated the use of this assembly to treat Pauwels type III femoral neck fractures in young adults (Gümüstas et al., 2014; Hoshino et al., 2016; Parker et al., 1991; Sirkin et al., 1999).

Some aspects must be analyzed for correct application of the surgical technique using the medial plate combined with cannulated screws. The first of these is the tendency for the femoral neck to rotate, which was observed after mechanical load was applied in step 1 of this experiment. We were unable to find an adequate explanation for this finding, although there was no failure in varus or shearing during step 2. The use of three screws parallel to the long axis of the femur neck instead of one horizontal screw (the Pauwels screw) may reduce this tendency since the presence of the medial plate increases compression force (converting the shear forces). The second is the presence of the medial plate very close to the articular capsule, or even within the capsule, as discussed by Mir and Collinge (Mir & Collinge, 2015). In the series by Ye et al., implant removal was not required in any patient for any reason, including implant proximity to the articular capsule (Ye et al., 2017). However, future studies will show if this concern is more theoretical or whether it deserves greater attention, and possibly may lead to adjustments to the surgical technique. The third is the potential risk of vascular damage to the femoral head, specifically the inferior retinacular artery, which was demonstrated to have a significant role in femoral head perfusion after femoral neck fractures (Lazaro et al., 2013). In a recent paper, Putnam et al. observed that the intraarticular course of the inferior retinacular artery lies within the Weitbrecht ligament between the femoral neck clock-face positions of 7:00 and 8:00, concluding that if the medial buttress plate is positioned at 6:00 along the femoral neck it will be anterior to the location of this artery and would not endanger the blood supply of the femoral head (Putnam et al., 2019).

This study has a number of limitations. First, the use of plastic bones, although this variable was controlled by the fact that the entire experiment was conducted using identical models from the same lot. Furthermore, a non-fracture control group was used to evaluate the mechanical resistance of the models and adjust the loads used in the experiment accordingly. Cristofolini et al. investigated the mechanical behavior of plastic models and found no significant differences in comparison with two groups of human femurs (fresh-frozen and freeze-dried/rehydrated) (Cristofolini et al., 1996). Finally, the use of plastic bone models presents an advantage over fresh or frozen human bones because the interfemoral variability of synthetic bones is 20 to 200 times less than in cadaverous specimens. This allows small differences to be characterized as significant, even with a small sample (Cristofolini et al., 1996). Secondly, was the lack of a comparative group using the sliding hip screw system (SHS), which is considered nowadays a more effective treatment for osteosynthesis of femoral neck fractures in the young patient (Ma et al., 2018). Nowotarski et al. and Hoshino et al. showed in biomechanical and cohort studies, respectively, that the use of hip-screw systems results in fewer fixation failures in Pauwels type III femoral neck fractures compared with constructions using only cannulated screws (Nowotarski et al., 2012; Hoshino et al., 2016). On the other hand, Gupta et al., in a clinical-radiological outcome study comparing cannulated screws with SHS in 85 young patients with displaced femoral neck fractures, found no significant difference between these two implants (Gupta et al., 2016). In this study, our objective was to observe the mechanical behavior of two recent fixation options for this fracture which employ screws alone, considering that few articles in the literature support the use of these techniques or describe their surgical techniques in greater detail (Augat et al., 2019). Based on the results of our experiment, which demonstrated the mechanical superiority of the medial plate associated with cannulated screws, future investigations may extend beyond comparison of this arrangement with fixed-angle angle systems. Thirdly, not all of the vectors of force which affect the hip during physiological muscle contraction activities were simulated. In an electromyographic study, Giordano et al. demonstrated that the force exerted by the gluteus medius-tractus iliotibialis does an excellent job of reproducing what occurs in the hip during single-leg support (Giordano et al., 2006). Nevertheless, the majority of the tests evaluating the mechanical resistance of fixation at the proximal end of the femur reproduce only the axial load vector, which results from the action of the gluteus medius muscle and body weight (Zdero et al., 2010; Aminian et al., 2007; Walker et al., 2007). Finally, no washers were used in the study model, despite their important role in both distributing force as well as preventing penetration of the screw head into the lateral cortex of the femur (Bishop et al., 2014; Zlowodzki et al., 2015). However, because we used plastic models that reproduce the bone mineral density of a femur in a young adult, there is less risk of the complications described above, which are more commonly observed in patients with osteoporosis (Zlowodzki et al., 2005). Furthermore, no washers were used in any of the specimens in the current study, which provided a homogeneous assessment.

## Conclusion

Biomechanical tests using synthetic bone models with a Pauwels type III femoral neck fracture demonstrated that fixation using two parallel inferior cannulated screws and a third more horizontal screw (the Pauwels screw) in combination with a medial side plate is superior to fixation using only the cannulated screws in the same arrangement. The mode of failure normally observed in this fracture pattern (varus and shear stress deviations) is not seen in the combined technique, probably because the medial plate converts shearing forces into compression forces, and particularly optimizes the function of the screws which are parallel to the axis of the femoral neck.
